# Evaluation of Composition Effects on the Physicochemical and Biological Properties of Polypeptide-Based Hydrogels for Potential Application in Wound Healing

**DOI:** 10.3390/polym13111828

**Published:** 2021-05-31

**Authors:** Johnel Giliomee, Lisa C. du Toit, Pradeep Kumar, Bert Klumperman, Yahya E. Choonara

**Affiliations:** 1 Wits Advanced Drug Delivery Platform Research Unit, Department of Pharmacy and Pharmacology, Faculty of Health Sciences, School of Therapeutic Sciences, University of the Witwatersrand, Johannesburg, 7 York Road, Parktown 2193, South Africa; johnel676@gmail.com (J.G.); lisa.dutoit@wits.ac.za (L.C.d.T.); pradeep.kumar@wits.ac.za (P.K.); 2 Department of Chemistry and Polymer Science, Faculty of Science, University of Stellenbosch, Private Bag X1, Matieland 7602, South Africa; bklump@sun.ac.za

**Keywords:** polypeptide-based hydrogel, wound healing, ring-opening polymerization, poly-l-lysine, 4-arm poly(ethylene glycol)

## Abstract

In this study, the effect of crosslinking and concentration on the properties of a new library of low-concentration poly(Lys_60_-*ran*-Ala_40_)-based hydrogels for potential application in wound healing was investigated in order to correlate the hydrogel composition with the desired physicochemical and biofunctional properties to expand the assortment of poly-l-lysine (PLL)-based hydrogels suitable for wound healing. Controlled ring-opening polymerization (ROP) and precise hydrogel compositions were used to customize the physicochemical and biofunctional properties of a library of new hydrogels comprising poly(l-lysine-*ran*-l-alanine) and four-arm poly(ethylene glycol) (P(KA)/4-PEG). The chemical composition and degree of crosslinking via free amine quantification were analyzed for the P(KA)/4-PEG hydrogels. In addition, the rheological properties, pore morphology, swelling behavior and degradation time were characterized. Subsequently, in vitro cell studies for evaluation of the cytotoxicity and cell adhesion were performed. The 4 wt% 1:1 functional molar ratio hydrogel with P(KA) concentrations as low as 0.65 wt% demonstrated low cytotoxicity and desirable cell adhesion towards fibroblasts and thus displayed a desirable combination of properties for wound healing application.

## 1. Introduction

The intricate process of wound healing is well understood [1,2,3,4]. This process includes the three overlapping phases of wound healing, namely, inflammation, proliferation and remodeling. These phases are carefully controlled by growth factors and cytokines. Understanding the interactive processes involved in each of these events has been essential to the development of new wound therapies. Multiple strategies to treating wounds currently exist [5,6,7]. These include biological dressings, active dressings and skin substitutes such as epidermal and dermal substitutes. However, the search for the ideal treatment continues. The ideal treatment should provide a combination of rapid wound closure, control over infections, prompt pain relief, minimal inconvenience to the patient and minimal scarring while maintaining low treatment costs and risks [8]. Advanced treatments, such as bioactive dressings and skin substitutes, aim to achieve these criteria by mimicking the native skin and actively partaking in the wound healing process [7,9,10,11,12]. Hydrogel wound dressings are particularly interesting [11]. Due to their ability to absorb 30–100% of water [13], they can maintain a moist wound environment favorable to the wound healing process. The similarities between hydrogels and the macromolecular components of the extracellular matrix (ECM) have also long been recognized [13,14]. Their highly porous hydrated matrix gives them the ability to support the incorporation of cells as well as active components such as drugs and biological molecules [11,15].

Polypeptide-based hydrogels have gained a particular interest in biomedical applications [16,17]. This has mainly been ascribed to their inherent biological properties and widely tunable physicochemical properties [17]. Compared to naturally derived materials, which typically suffer from batch-to-batch variation and difficult extraction methods, polypeptides can be synthetically produced with controlled chemical and physical properties [14]. Controlled ring-opening polymerization (ROP) is a widely reviewed technique used to produce polypeptides with a controlled molecular weight and molecular weight distribution, and advanced architectures from *N*-carboxy anhydride (NCA) amino acids [18,19]. The use of natural and synthetic amino acids allows the incorporation of biocompatibility, biodegradability, chemical functionality and specialized functionalities, such as transferring antimicrobial properties into polypeptides [20,21]. Polypeptides have been formed into functional hydrogels through a variety of physical or covalent crosslinking techniques [17]. The assembly of polypeptides into hierarchical and supramolecular structures drives the gelation of physical polypeptide-based hydrogels [17,22,23]. The functional moieties on the side chains of polypeptides also allow covalent crosslinking through a variety of chemical reactions [17].

Recently, a number of researchers have investigated the tissue engineering and wound healing potential of hydrogels composed of the well-known polypeptide poly (l-lysine) (PLL) and its copolymers. PLL is commonly used as a coating due to its cell adhesion properties. Contrary to PLL in its free polymer form, which is known to be cytotoxic to mammalian cells [24,25], PLL-based hydrogel networks have shown decreased cytotoxicity and even good biocompatibility [26]. The free amines on the l-lysine side chains afford easy modification and chemical crosslinking into hydrogel networks. The biocompatibility of such hydrogels is dependent on several factors. Hynes et al. [27] demonstrated the effect of the molecular weight of PLL and the amine content of the hydrogel on the proliferation and differentiation of neuronal stem cells. Pakstis et al. [26] reported on the role of the morphology of the self-assembled polypeptide in the hydrogel compared to its free morphology in solution. Song, Rane and Christman [28] also reported on the effect of the cationic to hydrophobic ratio of poly (Lys_x_-*ran*-Ala_y_) random copolymers crosslinked with multi-arm PEG into hydrogels. The hydrogels composed of poly(Lys_60_-*ran*-Ala_40_) showed good fibroblast cell adhesion and proliferation. The physical properties of hydrogels such as porosity, swelling, mechanical properties and biodegradability also play an important role in the potential tissue engineering applications [14,29]. It is further known that there is a complicated interplay between the physicochemical and biofunctional properties of hydrogels, and finding the ideal combination of these properties is desirable.

In this study, we investigated the effect of crosslinking and concentration on the properties of a new library of low-concentration poly(Lys_60_-*ran*-Ala_40_)-based hydrogels. The aim was to correlate the hydrogel composition with the desired physicochemical and biofunctional properties, in order to expand the assortment of PLL-based hydrogels suitable for wound healing. By controlling the synthesis of the polypeptide, using a facile method of controlled ROP, we were able to target the lysine to alanine ratio (K/A), molecular weight and a narrow molecular weight distribution. This allowed carefully controlling the degree of crosslinking using stoichiometric ratios of the functional groups of the polypeptide and the multi-arm PEG while maintaining low total polymer concentrations.

## 2. Materials and Methods

### 2.1. Materials

All chemicals and solvents were purchased from commercial sources and used without further purification unless stated otherwise. Dry solvents were used as received and handled under dry inert gas. Fmoc-l-Lys(Boc)-OH, l-alanine, triphosgene, benzylamine, α-pinene and trifluoroacetic acid (TFA) were purchased from Sigma Aldrich (St. Louise, MO, USA). Four-arm PEG-succinimidyl glutarate (4-PEG-SG) (10 kDa) was procured from JenKem Technology. SnakeSkin^®^ Pleated dialysis tubing (3500 MWCO) was purchased from Thermo Fischer Scientific. Moisture- and oxygen-sensitive reactions were carried out under an inert atmosphere using dry nitrogen (N_2_) gas. All compounds were characterized by ^1^H NMR spectroscopy using a Bruker AVANCE spectrometer (300 MHz or 500 MHz, Bruker, Germany). Samples were dissolved in deuterated solvents. Chemical shifts are reported in parts per million (ppm), where tetramethylsilane (TMS) is used as internal reference. Splitting patterns are designated as s (singlet), d (doublet), t (triplet), q (quartet) and m (multiplet), whereas coupling constants (J) are reported in hertz (Hz). Fourier transform infrared (FTIR) spectrometry was performed on a Spectrum 100 FT-IR Spectrometer (PerkinElmer) fitted with a Universal ATR Sampling Accessory. Size exclusion chromatography (SEC) was measured on a system equipped with a Shimadzu LC-10AT isocratic pump, a Waters 717+ autosampler, a column system fitted with a PSS guard column (50 × 8 mm) in series with three PSS GRAM columns (300 × 8 mm, 10 µm, 2 × 3000 Å and 1 × 100Å) kept at 40 °C, a Waters 2487 dual-wavelength UV detector and a Waters 2414 differential refractive index (DRI) detector. *N*,*N*-Dimethylformamide (DMF) was used as the eluent, stabilized with 0.05% BHT (*w*/*v*) and 0.03% LiCl (*w*/*v*), at a flow rate of 1 mL·min^−1^. Prior to analysis, polymer samples were filtered through 0.45 µm GHP filters. The molar masses of polymers were calculated against poly(methyl methacrylate) (PMMA) standards (Polymer Laboratories) ranging from 690 to 1.2 × 10^6^ g·mol^−1^.

### 2.2. Synthetic Procedures

#### 2.2.1. N_ε_-*tert*-Butyloxycarbonyl l-lysine (H-Lys(Boc)-OH)

H-Lys(Boc)-OH was prepared via the deprotection of Fmoc-Lys(Boc)-OH using a modification of a procedure described in the literature [30]. Fmoc-Lys(Boc)-OH (10.0 g, 21.3 mmol) was dissolved in 40 mL dimethyl sulfoxide (DMSO) and heated to 120 °C under reflux. The reaction mixture was stirred for 1 h while a white precipitate formed. The product was isolated by precipitation in diethyl ether. Repeated washing with diethyl ether and vacuum drying afforded H-Lys(Boc)-OH as a white powder. ^1^H NMR (300 MHz, DMSO) δ 6.75 (s, 1H), 3.07 (t, *J* = 6.10 Hz, 1H), 2.97–2.76 (q, 6.74, 2H), 1.75–1.44 (m, 2H), 1.44–1.15 (m, 13H).

#### 2.2.2. Synthesis of *N*-carboxyanhydride (NCA) of N_ε_-tert-butyloxycarbonyl l-lysine (NCA-Lys(Boc))

H-Lys(Boc)-OH (5.00 g, 20.3 mmol) and α-pinene (7.41 g, 54.4 mmol) were dissolved in dry THF (60 mL) in a 250 mL 3-neck round-bottom flask. The solution was heated under inert reflux conditions. Triphosgene (2.76 g, 9.3 mmol) was dissolved in dry THF (24 mL) and added dropwise to the reaction mixture once reflux started. The solution became clear during the addition of triphosgene and then started forming a white precipitate by-product. After 1 h, the reaction mixture was cooled, and the precipitate was removed by filtration. The mother liquor was reduced to a quarter of the volume. An amount of 20 mL *n*-heptane was added, and the solution was heated to recrystallize. The pure product was obtained by repeated recrystallizations from *n*-heptane and THF (58% yield). The product was then dried under vacuum and stored in a freezer under P_2_O_5_ and N_2_ gas. ^1^H NMR (500 MHz, DMSO-*d*_6_) δ 9.08 (s, 1H), 6.78 (s, 1H), 4.43 (t, *J* = 6.0 Hz, 1H), 2.90 (q, *J* = 6.4 Hz, 2H), 1.92–1.56 (m, 2H), 1.47–1.20 (m, 13H).

#### 2.2.3. Synthesis of *N*-carboxyanhydride (NCA) of l-alanine (NCA-Ala)

l-Alanine (5.00 g, 56.1 mmol) and α-pinene (17.58 g, 129 mmol) were dissolved in dry THF (60 mL) in a 250 mL 3-neck round-bottom flask. The solution was heated under inert reflux conditions. Triphosgene (11.16 g, 37.6 mmol) was dissolved in dry THF (24 mL) and added dropwise to the reaction mixture once reflux started. After 3 h, all solids had dissolved to leave a clear solution. The solution was reduced to a quarter of the volume and the same purification was followed as for NCA-Lys(Boc) (50%). ^1^H NMR (500 MHz, DMSO-*d*_6_) δ 8.98 (s, 1H), 4.48 (q, *J* = 7.0 Hz, 1H), 1.33 (d, *J* = 7.1 Hz, 2H).

#### 2.2.4. Random NCA Copolymerization

The targeted degree of polymerization (DP) was 100, with a monomer ratio of NCA-Lys(Boc) to NCA-Ala of 0.6:0.4. The polymerization was performed as follows: NCA-Lys(Boc) (2.00 g, 7.34 mmol), NCA-Ala (0.56 g, 4.89 mmol) and dry DMF (15 mL) were added to a Schlenk tube. The monomer solution was then subjected to four repeated freeze–pump–thaw cycles. In a separate vial, benzylamine (0.013 g, 0.12 mmol) and dry DMF (2.5 mL) were sparged with N_2_ gas. The benzylamine initiator solution was then added to the monomer solution under inert conditions. The final reaction mixture was cooled to 0 °C and placed under vacuum conditions. The reaction proceeded for 96 h at 0 °C. The protected polypeptide was isolated by precipitation from diethyl ether.

#### 2.2.5. Deprotection of Polypeptides

Deprotection of the polypeptide was performed using TFA according to literature procedures. The protected polypeptide (1.00 g) was dissolved in TFA (5.00 g) and stirred for 2 h. The polypeptide poly(Lys_x_-*ran*-Ala_y_) was then precipitated in diethyl ether. Repeated washing with diethyl ether afforded a powdery white product. This was further purified via dialysis, using a 3.5 kDA MWCO Snakeskin, against distilled water changed twice daily for three days.

### 2.3. Preparation of Polypeptide-Based Hydrogels

The polypeptide-based hydrogels (Table 1) were prepared with the synthesized polypeptide, entry #2 in Table 2 (further referred to as P(KA) for simplicity), crosslinked with 4-PEG-SG. Different molar ratios of the free amines from P(KA) to the NHS esters from 4-PEG-SG (1:1, 2:1, 4:1, 8:1 and 16:1) and different polymer concentrations (1 wt%, 2 wt%, 4 wt%, 6 wt% and 8 wt%) were used. Solutions of P(KA) and 4-PEG-SG were prepared in phosphate buffers of pH 9 and pH 4, respectively. Equal volumes of each were then added together to make the precursor hydrogel solution. This solution was then allowed to gel over time.

### 2.4. Hydrogel Characterization

#### 2.4.1. Gelation and Rheometry

The precursor hydrogel solutions (0.5 mL) were prepared in glass vials. After different time intervals, the vials were inverted to determine qualitatively if they were a solution or a gel phase. Gels were also manipulated with a spatula, and if it could be spread on the glass vial wall, the gel was designated “soft/gel-like”. If the gel retained its shape, it was designated “hydrogel”.

The gelation of selected hydrogel solutions was further investigated using a Thermo Scientific™ HAAKE™ MARS™ Rheometer (Thermo Fischer Scientific, Karlsuhe, Germany), with a 35 mm cone plate with a 1° incline and a 0.052 mm gap distance fitted with a HAAKE™ Universal Temperature Controller (Thermo Fischer Scientific, Karlsuhe, Germany). An amount of 0.2 mL of the precursor hydrogel solutions was added to the rheometer plate and allowed to gel. For time sweep experiments, the evolution of the storage modulus (G′) and the loss modulus (G″) from t_0_ was measured. A constant shear rate of 10 Pa at a frequency of 1 Hz was used to measure G′ and G″ over a time of 1500 s.

The time to equilibrium was determined from the time sweep and was implemented as a waiting time for subsequent tests. For amplitude sweeps, τ was increased from 0.01 to 1000 Pa at a constant frequency of 1 Hz, while for frequency sweeps, the frequency was increased from 0.01 to 100 Hz at a constant τ of 10 Pa.

#### 2.4.2. Amine Quantification

The amount of free amines present in the hydrogels was determined using a Kaiser assay against a standard concentration curve of H-Lys(Boc)-OH. A ninhydrin solution containing equal volumes of each component in a Kaiser Kit (Sigma Aldrich, St. Louise, MO, USA) was freshly prepared and used immediately. Freeze-dried samples of hydrogels were suspended in 1 mL of the prepared ninhydrin solution in test tubes. The test tubes were closed with aluminum foil and parafilm and incubated for 30 min at 80 °C. The test tubes were then left to cool to room temperature and 4 mL ethanol was added. The solutions were then filtered through 0.45 μm PVDF syringe filters. The UV absorbance was then measured at 570 nm on a UV spectrophotometer using a quartz cuvette with 10 mm path length.

#### 2.4.3. Pore Morphology

The pore sizes were analyzed using scanning electron microscopy (SEM). Hydrogel samples were swollen in deionized water and frozen at −80 °C overnight. Frozen samples were cut in half and lyophilized. The dried samples were mounted on stubs with the cross-section facing upwards. Samples were then coated with gold (Au) using an SPI™ Sputter Coater. SEM micrographs of the pores were taken using a FEI™ Phenom™ Desktop SEM (Phenom™, FEI Company, OR, USA). The images were analyzed using ImageJ software (version 1.51n), and the size distributions were calculated using 40 to 60 measurements from 3 or more images with size distributions approximating normal distributions.

#### 2.4.4. Swelling Behavior

The equilibrium swelling ratios (*ESRs*) of the “as-prepared” and “freeze-dried” hydrogels were determined from Equation (1):

(1) $$ESR=\frac{\left(W_{W}-W_{D}\right)}{W_{D}}$$

where *W_W_* is the wet weight of the hydrogel after swelling in PBS and *W_D_* is the dry weight of the hydrogel after freeze drying.

For the “as-prepared” *ESR*, the hydrogel sample was swollen for 18 h in deionized water after the gelation time. The swollen hydrogel was placed on filter paper to remove excess water and weighed. The weight was recorded over time until it remained constant. The final weight was then recorded as *W_W_*. The swollen hydrogel was frozen at −80 °C overnight and lyophilized. The weight of the dry hydrogel was then recorded as *W_D_*.

For the “freeze-dried” *ESR*, the dried hydrogel was swollen in PBS, and the *W_W_* was recorded as above.

#### 2.4.5. Degradation Time

Hydrolytic degradation of the freeze-dried hydrogels was performed in PBS (pH 7.2) at 37 °C with constant agitation from an orbital shaker and daily PBS solvent changes. Over 28 days, the weight of the hydrogels was recorded after drying the excess PBS on filter paper. If the hydrogel was visibly disintegrated, no weight was recorded, and the time was recorded as the degradation time in days.

### 2.5. In Vitro Cell Studies

Cytotoxicity and cell adhesion were tested on NIH 3T3 mouse fibroblast cells, using 4 wt% P(KA)/4-PEG hydrogels with molar ratios of 1:1 to 16:1. For the positive control, cells were cultured in fresh medium composed of Dulbecco’s Modified Eagle Medium (DMEM) supplemented with 10% fetal bovine serum and 1% Penicillin-Streptomycin solution (10,000 units penicillin and 10 mg streptomycin/mL) to a seeding density of 7500 cells/well, while in the negative control, cells were treated with DMSO.

#### 2.5.1. Cytotoxicity

Cells (100 μL) in fresh medium (7500 cells/well) were added to 96-well plates and incubated for 24 h with 5% CO_2_ at 37 °C. Hydrogel discs were prepared by placing 20 μL of the precursor hydrogel solutions between a microscope slide and cover slip, along with a 1 mm spacer, and allowed to gel for 1 h. The hydrogels were prepared under sterile conditions, washed with PBS (pH 7.2) and swollen in fresh media. They were then added onto the seeded cell layers and incubated for 24 h. The hydrogel disks were then carefully removed, without disturbing the cell layer, and 20 μL of a 3-(4,5-dimethylthiazol-2-yl)-2,5-diphenyltetrazolium bromide (MTT) solution was added to each well and incubated for 4 h. The media in each well were then aspirated, 100 μL DMSO was added and the plate was placed on an orbital shaker for 40 min to dissolve the formazan crystals. The supernatant from each well was then transferred to a clean plate, and the absorbance was measured at 570 nm on a Victor™ X3 2030 Multilabel Reader (PerkinElmer Ltd., Beaconsfield, UK).

#### 2.5.2. Cell Adhesion

The precursor hydrogel solutions (70 μL) were added to the 96-well plate and allowed to gel for 1 h. Hydrogels were then washed with PBS (pH 7.2) and swollen in fresh media. NIH 3T3 cells (100 μL) in fresh medium (7500 cells/well) were added to the prepared hydrogels and cultured for 48 h at 37 °C with 5% CO_2_. The cellular adhesion was qualitatively evaluated using a CKX53 light microscope from Olympus® (Shinjuku, Tokyo, Japan).

### 2.6. Statistical Evaluation

Results are reported as mean ± standard deviation. One-way analysis of variance (ANOVA) and Student’s *t*-test were used for comparison between groups. A value of *p* < 0.05 was considered statistically significant.

## 3. Results and Discussion

### 3.1. Preparation of Controlled Polypeptides

**Scheme 1:**
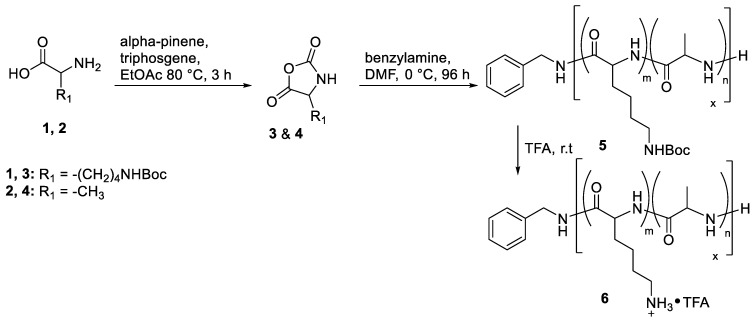
Synthesis of NCA amino acids and copolymerization via ROP.

The NCA monomers NCA-Lys(Boc) and NCA-Ala were produced according to literature procedures where triphosgene was used with α-pinene as a HCl scavenger (Scheme 1) [31]. Recrystallization was used to produce the NCA monomers in high purity. Removal of impurities such as *N*-chloroformyl amino acid chlorides and α-isocyanato acid chlorides was carefully considered since they are known to influence the ability to control the subsequent ROP [19,32].

Control over any living chain-growth polymerization, also just referred to as living polymerization, requires initiation primarily from the propagating chain, while transfer and termination reactions remain absent [33]. NCA ROP is conventionally initiated via nucleophiles such as primary amines. These polymerizations are, however, highly susceptible to side reactions and termination reactions and are therefore difficult to control [18,19]. Strategies to control NCA ROP initiated with primary amines include the use of high-purity chemicals and vacuum techniques [32]. It was also reported that low reaction temperatures can reduce the side reactions and afford living polymers suitable for advanced copolymer synthesis [31,34,35]. This eliminates the need for specialized polymerization techniques using transition metal catalysts, organocatalytic systems, ammonium halide initiators and silazane mediators [18]. In this study, the synthesis of the polypeptide copolymer poly(Lys_m_-*ran*-Ala_n_) was controlled using literature procedures, where benzylamine was used as an initiator at 0 °C (Scheme 1) [35]. A monomer ratio of 0.6 to 0.4 (K/A) and a total monomer to initiator ratio (M/I) of 100 were used to target the desired polypeptide composition, poly(Lys_60_-*ran*-Ala_40_), as reported by Song, Rane and Christmane [28].

**Figure 1:**
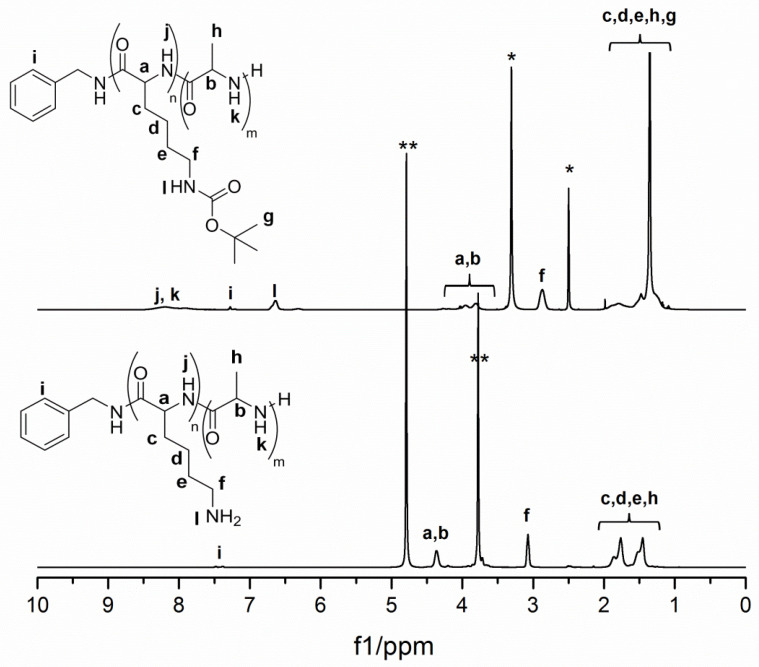
^1^H NMR spectra of K_46_A_33_ before (top) and after (bottom) deprotection of the lysine residues. Asterisk (*) indicates residual solvent peaks from d^6^-DMSO (d^5^-DMSO and HDO) and double asterisks (**) indicate residual solvent peak from D_2_O (HOD) [36].

Since no further chain extension after the initial polymerization was required, it is adequate to assess the number average molar mass (*M*_n_), dispersity (*Đ*) and the K/A molar ratio as a measure of the control over the polymerization. The expected M_n_ determined from conversion was not possible from ^1^H NMR spectroscopy due to overlapping between monomer and polymer signals. Instead, the targeted M_n_ was correlated against the experimental M_n_ determined from ^1^H NMR spectroscopy and SEC (Table 2). From ^1^H NMR, the degree of polymerization (DP) and the K/A molar ratio can be used to calculate M_n_ using Equation (2):

(2) $$M_{n,\;\;\;NMR}=DP_{NMR}\left(x_{K}M_{K}+x_{A}M_{A}\right)+M_{I}$$

where *x_K_* and *x_A_* are the respective molar fractions of H-Lys(Boc)-OH and l-alanine, and *M_K_*, *M_A_* and *M_I_* are the molar masses of H-Lys(Boc)-OH, ʟ-alanine and benzylamine, respectively. The *DP* was determined from the ratio between the integrated benzyl chain end signal at **i** and the integrated polypeptide backbone signal at **a,b** in Figure 1. It should be noted that this calculation of the DP is based on the assumption that all chains were initiated by benzylamine. ^1^H NMR spectroscopy was further used to determine the K/A molar ratio of poly(Lys_x_-*ran*-Ala_y_). K/A was calculated from the protected poly(Lys(Boc)_x_-*ran*-Ala_y_), where signal **f** in Figure 1 was integrated against the combined signal **a,b**. Signal **f** corresponds to the two CH_2_ protons from K, while signals **a** and **b** correspond to the CH backbone protons from both K and A. The experimental ratios correlate reasonably well with the targeted ratio of 0.6:0.4, as summarized in Table 2. The lower DP from entry #1 compared to entry #2 can be ascribed to differences in the polymerization rate or loss of control over the polymerization. Based on the higher *Đ* obtained for entry #1 compared to entry #2, it is evident that slightly less control over the polymerization of entry #1 was obtained. Loss of control can be from initiation from moisture traces and from side reactions that can take place during the polymerization of NCA monomers [31]. The discrepancy between *M*_n, NMR_ and *M*_n, SEC_ can be expected since the SEC calibration was based on poly (methyl methacrylate) (PMMA) standards. Therefore, *M*_n_ and *Ð* values are not the true values for poly(Lys_x_-*ran*-Ala_y_) but, instead, are relative to PMMA standards.

The resultant poly(Lys(Boc)_x_-*ran*-Ala_y_) was deprotected using TFA and assessed using ^1^H NMR spectroscopy. In Figure 1, it is seen that the large signal at ~1.3 ppm, corresponding to the 9 CH_3_ protons from the Boc group, is absent after deprotection. The free amines in the form of TFA salts were further quantified using the Kaiser assay (Table 2). This colorimetric assay is well known for the quantification of amino acids, peptides and proteins [37]. It makes use of the formation of a purple dye complex, also known as Ruhemann’s purple, when ninhydrin reacts with primary amines. The amount of free amines was then quantified from UV/Vis spectroscopy using a standard curve of H-Lys(Boc)-OH. The amount of free amines was further used to calculate the molar ratios for the preparation of the hydrogels.

### 3.2. Preparation of a Library of Hydrogels

**Scheme 2:**
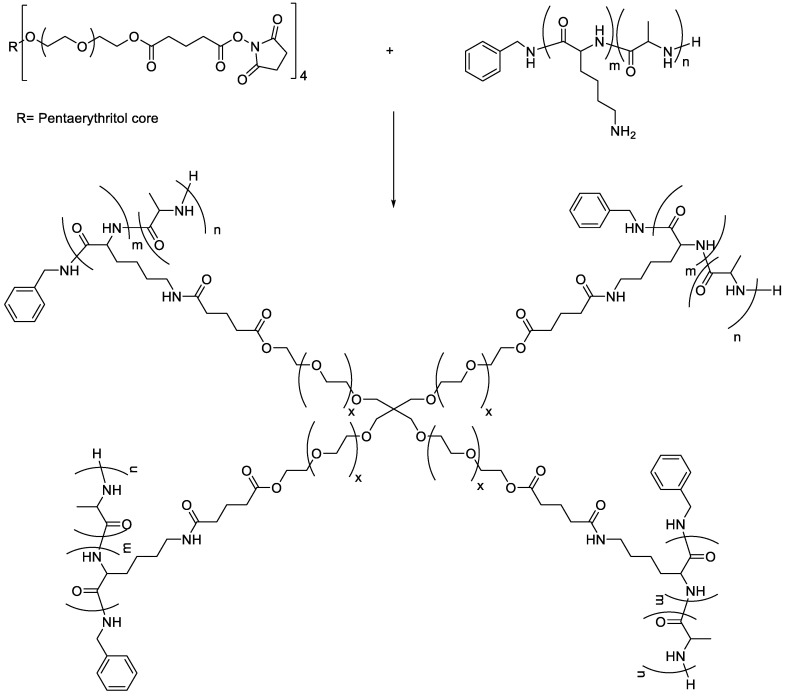
Crosslinking reaction between P(KA) and 4-PEG forms a covalent hydrogel network, P(KA)/4-PEG and NHS by-product.

**Figure 2:**
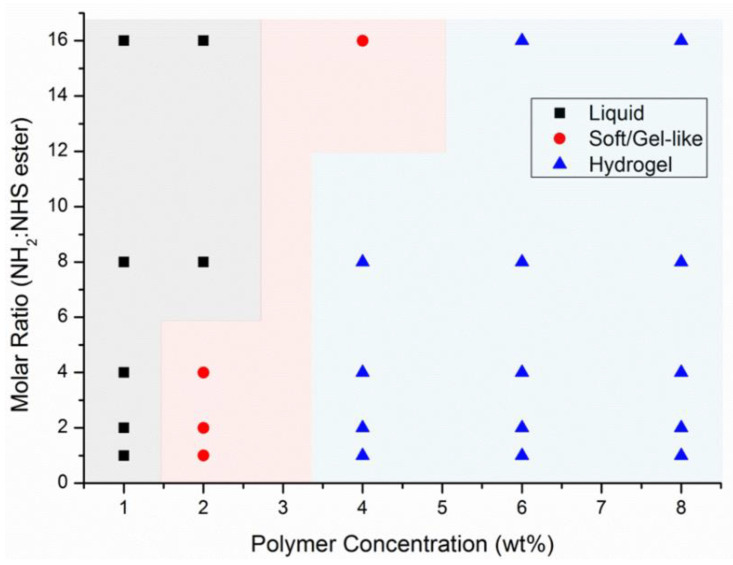
A phase plot of the library of hydrogels.

The reaction between primary amines and *N*-hydroxysuccinimide (NHS) esters is a well-known acylation reaction, where an amide is produced with the release of an NHS leaving group. In bioconjugation reactions, NHS esters are widely used to react with the α-amines at the *N*-terminus of proteins and peptides, and with the ε-amines of lysine side chains [38]. While the deprotonation of amines at higher pH will increase their reactivity, the hydrolysis of NHS esters also significantly increases at higher pH. Therefore, reactions between NHS esters and amines are typically performed under physiological conditions, where the hydrolysis rate of NHS esters and the reactivity of the amines are optimal. In this study, the polypeptide further referred to as P(KA) had ~60 amine functional groups, while 4-PEG-SG had four NHS ester functional groups. When the average effective functionality is >2, crosslinking can occur. This crosslinking can lead to the formation of an infinite network of polymers (Scheme 2). A library of P(KA)/4-PEG hydrogel solutions was prepared by varying the functional molar ratio of amines from P(KA) to succinimidyl esters from 4-PEG-SG, while the total polymer concentration was kept constant at 1 wt%, 2 wt%, 4 wt%, 6 wt% and 8 wt% (Table 1). It is known that the polymer concentration affects the crosslinking, with higher concentrations favoring intermolecular crosslinking and lower concentrations favoring intramolecular crosslinking [39]. In the reaction between P(KA) and 4-PEG-SG, complete intermolecular crosslinking will see each of the four arms of 4-PEG-SG react to a different P(KA) polymer molecule. In intramolecular crosslinking, two or more of the 4-PEG-SG arms will react with the same P(KA) polymer molecule. Therefore, intermolecular crosslinking favors the formation of an infinite gel network, while intramolecular crosslinking favors the solution phase. A higher polymer concentration and a functional molar ratio closer to 1:1 are expected to lead to a more densely formed polymer network, and the likelihood of forming a hydrogel will increase [40]. The vial inversion test was used to qualitatively identify the solutions as either liquid, gel-like or hydrogel [41]. This allowed the construction of a phase plot, which provided a visual presentation of how the molar ratio and concentration controlled the sol–gel transition of P(KA)/4-PEG (Figure 2). A clear phase transition was observed as the total polymer concentration increased from 1 to 4 wt%. A change in phase from liquid to hydrogel was not necessarily observed over the range of selected molar ratios. This is likely due to the high effective functionality of P(KA) and 4-PEG-SG, still favoring intermolecular crosslinking even as the functional molar ratio shifts from equimolar ratios. The materials designated as gel-like were noted as the sol–gel transition. These were found to be sticky and pliable with a spatula, contrary to the materials designated as hydrogels that maintained their 3D shape. It is likely that the number of intermolecular crosslinks, compared to intramolecular crosslinks, was too little to form a stiff gel. Critical gelation concentrations below 10 wt% are generally considered as low [42,43]. Low-concentration hydrogels are attractive for tissue engineering applications as they constitute lower toxicity and higher porosity. In this study, the concentration of P(KA) was varied from as little as 0.65 to 6.04 wt% for the hydrogels with total polymer concentrations of 4 wt%, 6 wt% and 8 wt%. Due to their robust nature, these hydrogels were selected for further investigation.

### 3.3. Assessment of the Rheological Nature of Selected P(KA)/4-PEG Hydrogels

**Figure 3:**
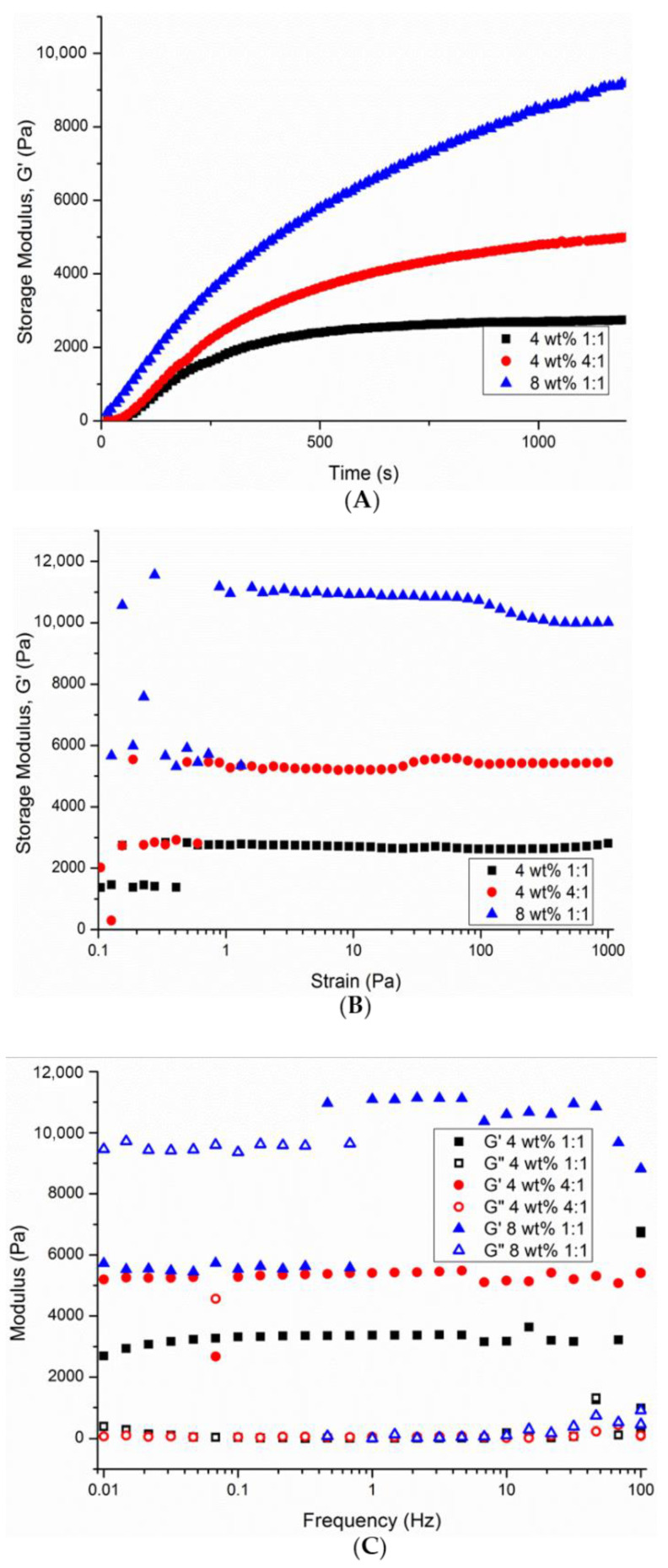
Rheological analyses of P(KA)/4-PEG hydrogels with 4 wt% (1:1 and 4:1 molar ratios) and 8 wt% (1:1 molar ratio) under (**A**) time sweep, (**B**) strain sweep and (**C**) frequency sweep settings.

Small-amplitude oscillatory shear (SAOS) was used to characterize the gelation time and equilibrium modulus of selected P(KA)/4-PEG hydrogels. Time sweeps were performed immediately upon mixing the two hydrogel components, and the evolution of the storage modulus (G′) and loss modulus (G″) over time was recorded (Figure 3A). The gelation of the 4 wt% hydrogel with a 1:1 molar ratio reached stability at 8 min near 2400 Pa, while the 4:1 molar ratio hydrogel reached stability at 15 min near 4800 Pa. While the 4:1 molar ratio hydrogel had a lower degree of crosslinking, it also had a much higher P(KA) content (Table 1), compared to the 1:1 molar ratio hydrogel. This could explain the higher stiffness of the hydrogel in its native form. The gelation of the 8 wt% hydrogel with a 1:1 molar ratio reached stability in 25 min near 10,000 Pa. This correlates with the expected trend that higher-concentration polymers will lead to a stiffer hydrogel. For subsequent tests, gels were allowed to reach equilibrium. Strain sweeps were performed to ensure that the moduli are recorded within the linear viscoelastic regime (LVE) [44]. Within this region, the moduli are independent from the amplitude of the deformation. For the three tested hydrogels, a strain of 10 Pa was within the LVE (Figure 3B). Frequency sweeps were further used to monitor the frequency dependence of the moduli. For gels, the equilibrium shear modulus (G_e_) is typically recorded at lower frequencies, where tan δ (also G″/G′) is <1 [44]. G_e_ was ~3400 Pa for the 4 wt% 1:1 molar ratio hydrogel, ~5400 Pa for the 4 wt% 4:1 molar ratio hydrogel and ~11,000 Pa for the 8 wt% 1:1 molar ratio hydrogel at 1 Hz (Figure 3C).

It is well known that the mechanical properties of hydrogels are closely related to cell behavior such as adhesion, proliferation and migration [45]. Dermal fibroblasts have been shown to migrate on a 3D gradient-based collagen hydrogel from nearly 1 to 2 MPa elastic modulus regions [46]. Nonetheless, natural biomaterials such as collagen, fibrin, elastin, gelatin and hyaluronic acid with generally low stiffness are widely used as skin dressings and skin substitutes [47]. The stiffness values displayed by the P(KA)/4-PEG hydrogels correlate reasonably well with similar materials indicated for soft tissue engineering [27].

### 3.4. Chemical Analysis of the Hydrogel Compositions

**Figure 4:**
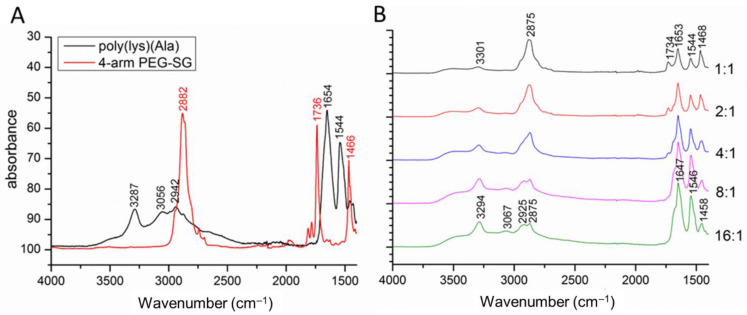
FTIR spectra of (**A**) starting materials and (**B**) hydrogels of 8 wt% with varying molar ratios.

FTIR analysis was used to provide qualitative information on the chemical compositions of the library of P(KA)/4-PEG hydrogels. The most notable IR bands from P(KA) observed in Figure 4 were assigned according to the well-documented bands from PLL [48,49]. This includes the ν_1_ proton mode band of the peptide group at 3287 cm^−1^, the ν_1_ band of the side chain NH_3_^+^ at 3056 cm^−1^, the amide I CO group stretching band at 1654 cm^−1^ and the amide II band from the in-plane deformational mode of the NH group at 1544 cm^−1^ [49]. The position of the latter two bands is supportive of the mainly random coil conformation of P(KA). The ν_1_ band of the side chain NH_3_^+^ was used by Rozenberg and Shoham [49] to estimate the relative content of protonated side chain NH_3_^+^ groups in samples isolated from different pH solutions. The relative intensity of this band for P(KA) with respect to the ν_1_ proton mode band of the peptide group is seen to decrease for P(KA)/4-PEG. Such a decrease in the relative content of NH_3_^+^ groups could be due to the crosslinking with NHS esters. The change in intensity of IR bands of P(KA) and 4-PEG from the P(KA)/4-PEG spectra correlates with the changing composition of the hydrogels as the molar ratio was increased from 1:1 to 16:1.

**Figure 5:**
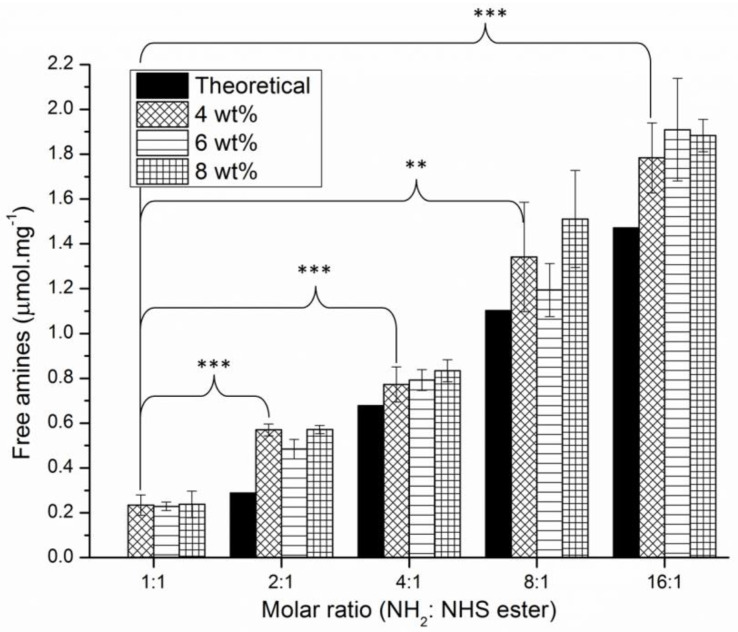
Concentration of free amines in the hydrogels compared to the calculated theoretical concentration when assuming complete reaction between the free amines and NHS esters. The effect of the molar ratio was found to be statistically significant as determined from Student’s *t*-tests. This is indicated by the double asterisks (**) for *p* < 0.01 and the triple asterisks (***) for *p* < 0.001 for the 4 wt% hydrogels. The effect of hydrogel concentration on the concentration of free amines was not statistically significant.

The degree of crosslinking of the P(KA)/4-PEG hydrogels was further investigated by quantifying the free amines present in the hydrogel network after crosslinking. By using the Kaiser assay, the formation of Ruhemann’s purple complex allowed colorimetric quantification of the amines even though the hydrogels are insoluble [50]. However, longer incubation times were necessary to release the purple complex from the hydrogel to the surrounding solution. The concentration of free amines was calculated based on the weights of freeze-dried hydrogels. The effect of the hydrogel concentration on the concentration of the free amines was not statistically significant, while the effect of the molar ratio of the hydrogels on their free amine concentration was found to be statistically significant (*p* < 0.05). The concentration increased from ~0.2 μmol·mg^−1^ for the 1:1 molar ratio hydrogels to ~2.0 μmol·mg^−1^ for the 16:1 molar ratio hydrogels. This can be compared to the theoretical free amines present when assuming a complete reaction between the amines and NHS esters. It is observed from Figure 5 that the amounts of free amines present after crosslinking follow the theoretical trend, indicating reasonable control over the preparation of the hydrogels. It is, however, seen that the experimental values are slightly higher compared to theoretical values. The theoretical values were calculated based on the assumption that each molecule of 4-PEG has four NHS ester end groups. However, NHS ester hydrolysis prior to the reaction was not taken into account. It is also expected that the hydrolysis of NHS esters will compete with the acylation reaction [38]. Furthermore, experimental parameters such as the time elapsed before mixing the 4-PEG solution with the P(KA) solution and their mixing rate will play a role in the crosslinking. A noticeable increase in the variance of the data of the 8:1 and 16:1 hydrogels may indicate a higher sensitivity of the crosslinking reaction to the experimental parameters as the concentration of NHS esters becomes very low.

### 3.5. The Effect of Crosslinking and Concentration on the Hydrogel Network

The equilibrium swelling theory of Flory and Rehner can be used to determine certain properties of the hydrogel network structure, including the average molar mass between crosslinks (*M_c_*), crosslinking density (*ρ_c_*) and mesh size (*ξ*) [51,52]. The calculations for *M_c_, ρ_c_* and *ξ*, as derived from the original theory by Flory and Rehner, require certain polymer parameters which are unknown for the P(KA)/4-PEG hydrogels. However, determining these parameters was deemed beyond the scope of this study. Nonetheless, according to the theory, M_c_ is proportional to the equilibrium swelling ratio (*ESR*). Investigating the *ESR* and hydrogel morphology provided adequate insight into the effect of crosslinking and the polymer concentration.

**Figure 6:**
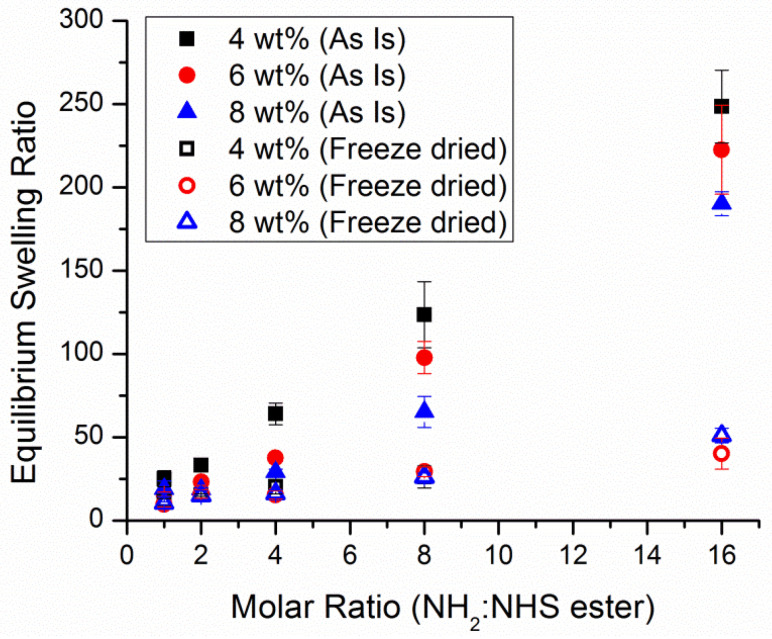
Equilibrium swelling of “as-prepared” and freeze-dried hydrogels as a function of NH_2_/NHS ester molar ratio is shown for 4 wt%, 6 wt% and 8 wt% hydrogels. The effect of molar ratio on the *ESR* was statistically significant for both “as-prepared” and freeze-dried hydrogels, as determined from Student’s *t*-tests (*p* < 0.05). The effect of hydrogel concentration was only statistically significant for the “as-prepared” hydrogels (*p* < 0.05).

The *ESR* of the library of hydrogels was investigated in their “as-prepared” and freeze-dried forms in deionized water. Freeze drying is widely used to induce the formation of micro-scale porous networks for tissue engineering applications [53,54,55,56,57]. Such micro-scale porosity is essential to promote cell infiltration, and mass transfer of nutrients and cellular waste [53]. The freezing process causes the formation of ice crystals, and a subsequent phase separation of the water and the polymer network. As a result, the polymer network is condensed into membrane-like structures. This can affect the native structure of the hydrogel and lead to differences between the *ESR* of the “as-prepared” and freeze-dried hydrogels. The effect of crosslinking and concentration on the *ESR* is visualized in Figure 6. An increase in the molar ratio of P(KA)/4-PEG hydrogels caused an increase in *ESR*. As expected, the lower degrees of crosslinking will allow more expansion of the polymer network [52]. In the “as-prepared” hydrogels, the ESR increased approximately 10-fold over the range of molar ratios, while for the freeze-dried hydrogels, the *ESR* only increased 5-fold. This increase in ESR as a function of the molar ratio was found to be statistically significant (*p* < 0.05) for both “as-prepared” and freeze-dried hydrogels. Furthermore, the effect of polymer concentration played a statistically significant role in the *ESR* of the “as-prepared” hydrogels (*p* < 0.05). The increase in polymer concentration caused a decrease in *ESR*. The higher polymer concentration presumably caused a denser polymer network less able to expand [51]. This further correlates with a larger intermolecular versus intramolecular crosslinking. However, this effect was statistically insignificant in the freeze-dried hydrogels (*p* > 0.05).

**Figure 7:**
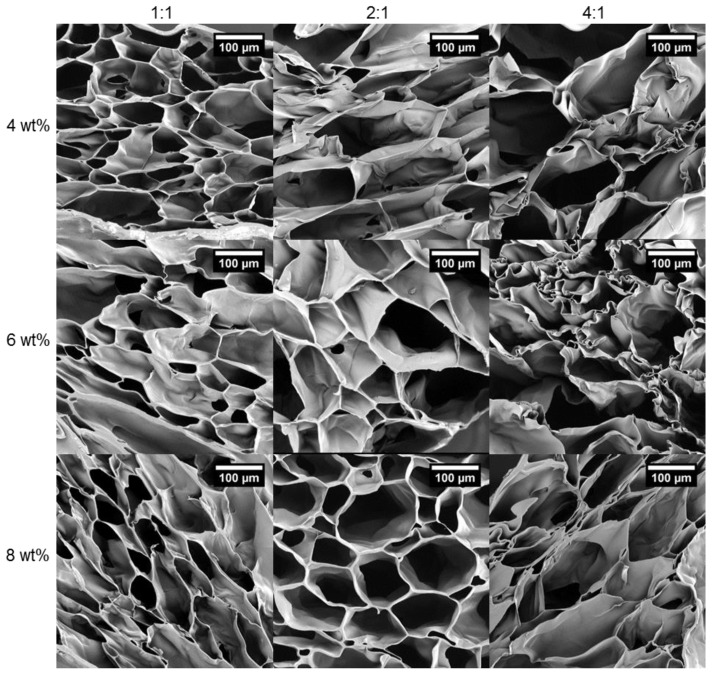
SEM images of the internal morphology of freeze-dried hydrogels.

The morphology of the freeze-dried hydrogels was investigated using SEM. Uniform interconnected pores separated by membrane-like structures were seen on the inner structure of the freeze-dried P(KA)/4-PEG hydrogels (Figure 7). A surface skin at the scaffold–air interface is commonly formed in freeze-dried hydrogels as a result of interface tension [58]. The higher molar ratios of 8:1 and 16:1 produced almost completely collapsed freeze-dried skins. Therefore, viewing the morphology of their inner structures was not possible. Their inner membranes were most likely too weak to support the 3D structure after freeze drying, due to the low degree of crosslinking. Evidence of this can be seen in Figure 7, where the inner membranes become less rigid and slightly collapsed as the molar ratio is increased from 1:1 to 4:1. While it was difficult to determine the actual pore sizes from such deformed structures, pore sizes were measured as they appear on the images (Table 3). Nonetheless, the results correlated with the *ESR* findings. A significant increase in the average pore size was observed as the molar ratio was increased from 1:1 to 4:1 (*p* < 0.05). The larger pore sizes correlate with a lower degree of crosslinking. This effect from the molar ratio seems dependent on the total polymer concentration, as it becomes less significant on the 8 wt% hydrogels. Similarly, the effect of polymer concentration on the morphology of the hydrogels seemed closely related to the molar ratio. An increase in the polymer concentration caused a statistically significant decrease in pore size for the 2:1 and 4:1 molar ratio hydrogels (*p* < 0.05), while it remained insignificant for the 1:1 molar ratio hydrogels. The smaller pore sizes were related to the denser polymer network of the higher-concentration hydrogels. The smaller size range of the 1:1 hydrogels corresponds with the optimal pore size range of 20–125 μm reported for the regeneration of adult mammalian skin [53].

### 3.6. Biocompatibility Assessment of the Hydrogels

**Scheme 3:**
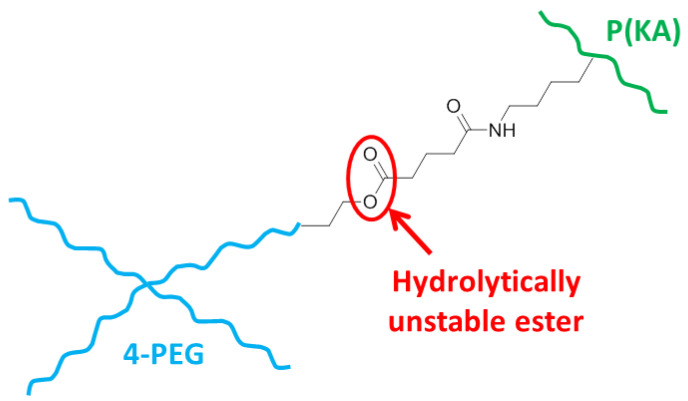
Schematic representation of the crosslinked P(KA)/4-PEG with the hydrolytically unstable ester encircled.

**Figure 8:**
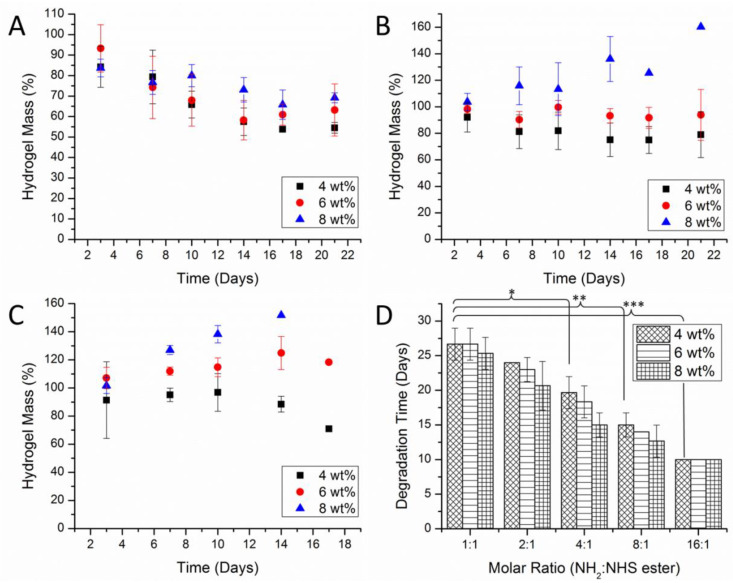
The effect of hydrolytic degradation on the hydrogel mass for (**A**) 1:1, (**B**) 2:1 and (**C**) 4:1 molar ratio P(KA)/4-PEG hydrogels, and (**D**) the effect of molar ratio on the final degradation time of the P(KA)/4-PEG hydrogels is shown. The effect of molar ratio was found to be statistically significant as determined from Student’s *t*-tests. This is indicated by the single asterisk (*) for *p* < 0.05, the double asterisks (**) for *p* < 0.01 and the triple asterisks (***) for *p* < 0.001 for the 4 wt% hydrogels. The effect of concentration on the final degradation time was not statistically significant.

The *in vitro* biocompatibility of the hydrogels was assessed in terms of degradation, cytotoxicity, and cell adhesion. The hydrolytic degradation of the hydrogels was investigated in a PBS buffer (pH 7.2). The remaining ester bonds in the spacer between the crosslinked 4-PEG and P(KA) are known to be hydrolytically cleavable (Scheme 3) [59]. Cleavage of these bonds can lead to the eventual dissolution of the hydrogel as the polymer network becomes disintegrated. The change in hydrogel mass as a function of time is often investigated as a measure of the disintegration of the hydrogel [60]. However, varying effects of crosslinking and concentration on the mass of the P(KA)/4-PEG hydrogels as they degraded provided little insight into the actual rate of degradation. For the 1:1 molar ratio hydrogels, a gradual decrease in hydrogel mass was observed, while for the higher-molar ratio hydrogels, a combination of mass increases and decreases was seen (Figure 8A–C). It can be expected that as the crosslinks are cleaved, more flexibility in the polymer network can also lead to higher swelling degrees and therefore an increase in the hydrogel weight.

In addition, the final degradation time of the P(KA)/4-PEG hydrogels was determined as the time when the hydrogel was visually disintegrated and unable to be weighed. In Figure 8D, the degradation time is seen to decrease from ±26 to ±10 days with the increasing molar ratio. The effect of the molar ratio on the degradation times was found to be statistically significant (*p* < 0.05). This followed the expected trend that hydrogels with lower degrees of crosslinking will degrade faster. However, the effect of the polymer concentration was statistically insignificant (*p* > 0.05). The relatively short degradation times correspond well with the rate of normal wound healing. Regeneration of the dermal and epidermal layers typically takes place within 21 days [6]. Therefore, the 1:1 and 2:1 molar ratio P(KA)/4-PEG hydrogels will be able to provide continued support to the healing wound while being gradually resorbed into the regenerated skin tissue.

**Figure 9:**
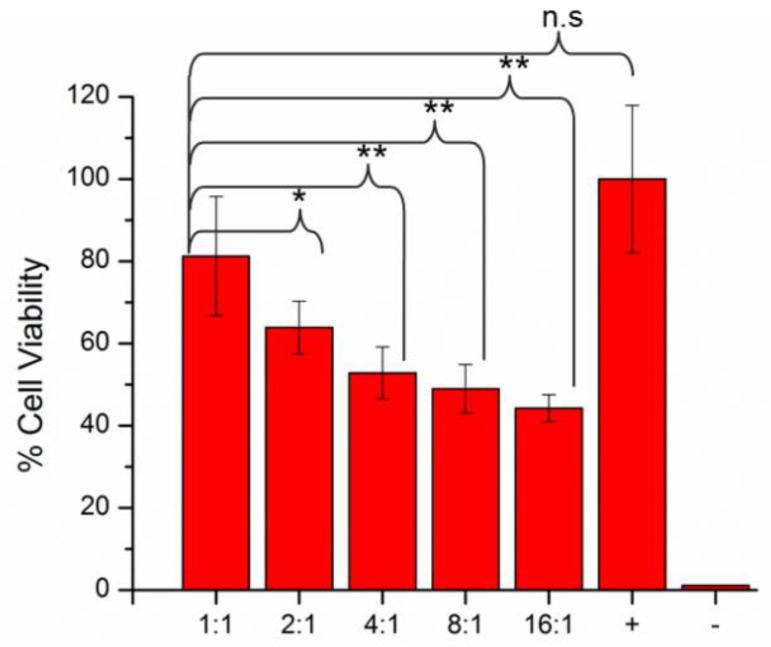
The effects of P(KA)/4-PEG hydrogel molar ratio on cell viability of NIH 3T3 cells are shown. The statistical significance is indicated by an asterisk (*) for *p* < 0.05 and double asterisks (**) for *p* < 0.01, as calculated from Student’s *t*-tests. The negative control group is represented by (−). The difference between the positive control group (+) and the 1:1 molar ratio group was not significant (n.s).

The cytotoxicity of the selected P(KA)/4-PEG hydrogels towards NIH 3T3 fibroblasts was evaluated using the well-known MTT assay. Viable cells metabolize MTT into purple formazan crystals, which can be solubilized, and the concentration can be quantitatively measured using UV spectroscopy. The cytotoxicity is therefore indirectly determined based on the number of metabolically active cells in reference to control groups. It is evident from Figure 9 that hydrogels prepared with a higher molar ratio caused a statistically significant decrease in the % cell viability (*p* < 0.05). The difference in % cell viability between the positive control group and the 1:1 molar ratio hydrogels was insignificant. From these results, it can be reasoned that the cytotoxicity of the 1:1 molar ratio hydrogels was adequately low to be further evaluated for tissue engineering applications.

**Figure 10:**
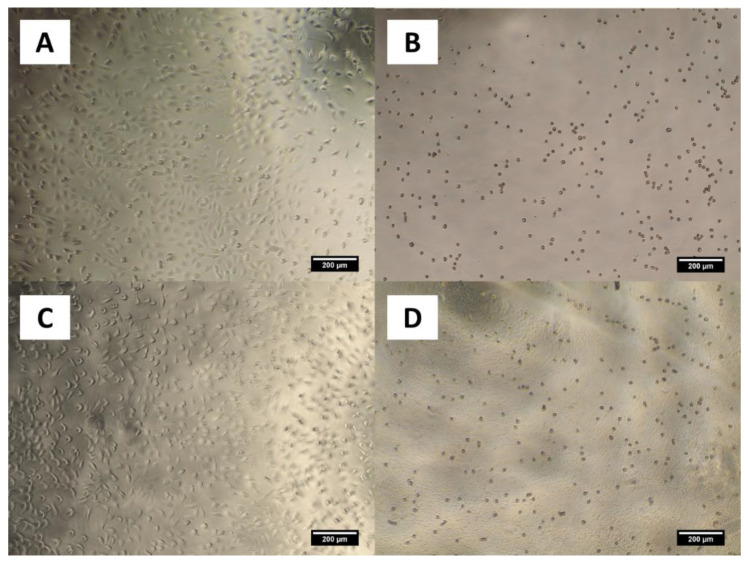
Images of T3T NIH cell adhesion on (**A**) well plate, (**B**) well plate with DMSO, (**C**) P(KA)/4-PEG hydrogel (4 wt%; 1:1 molar ratio) and (**D**) P(KA)/4-PEG hydrogel (4 wt%; 8:1 molar ratio) on light microscope (scale bar = 200 μm).

In addition to the cytotoxicity study, the cellular adhesion to the surface of the P(KA)/4-PEG hydrogels with the 4 wt% polymer concentration was assessed using light microscopy. Due to the transparent nature of the hydrogels, it was possible to view the morphology of the cells on the surface of the hydrogels. An elliptical, stretched cellular morphology is indicative of good cellular adhesion of fibroblasts, while poor adhesion is characterized by a spherical cellular morphology [28]. The morphology of the cells on the P(KA)/4-PEG hydrogel with a 1:1 molar ratio indicates good cellular adhesion, similar to the positive control (Figure 10). The P(KA)/4-PEG hydrogels with 2:1, 4:1 and 8:1 all showed poor cell adhesion. As an example, the cell morphology of the 8:1 hydrogel in comparison with the negative control (DMSO) is depicted in Figure 10. An increase in cellular debris was also noted as the molar ratio was increased, indicating potential cell membrane disruption.

The cytotoxicity of cationic polypeptides has been ascribed to the electrostatic interaction with anionic phospholipids in the cell membrane, causing membrane disruption and cell death [61]. Fischer et al. [24] investigated the cytotoxicity of different polycations, including PLL towards fibroblasts. They identified molecular weight and charge density as the main factors influencing the interaction of the polycations with the cell membrane. Hydrogels of PLL and its copolymers have lower cytotoxicity than the free polymers, most likely due to the decrease in charge density [27,62]. The charge density is reported as being dependent on the number of cations, as well as the three-dimensional structure and flexibility of the macromolecules. Hynes et al. [27] identified PLL-based hydrogels with free amine concentrations higher than 3 μmol/mg as cytotoxic to NSC cells, while hydrogels with lower-concentration amines maintained the cell viability. As discussed earlier, the concentration of free amines in the P(KA)/4-PEG hydrogels increased as the molar ratio increased. Therefore, the charge density of P(KA) is expected to increase, and the cytotoxicity towards the 3T3 cells is seen to follow the anticipated trend. Strategies to lower the charge densities on the high-molar ratio P(KA)/4-PEG hydrogels can include modifying the free amines through chemical conjugation [27].

## 4. Conclusions

The facile synthesis of P(KA) using controlled ROP afforded well-controlled polypeptides with narrow molecular weight distributions. The development of a library of P(KA)/4-PEG hydrogels allowed investigation into the effects of reactant ratios and the overall polymer concentration during gel formation on the physicochemical and biofunctional properties of these hydrogels. It was found that the properties of the hydrogels could be tuned significantly. This included the gelation time, mechanical properties, amine content, swelling behavior, pore sizes, degradation time and cell behavior. The cell behavior was found to be particularly sensitive to the molar ratio of the components in the hydrogels. The higher free amine concentration can be associated with a higher positive charge density and provided an explanation for the increase in cytotoxicity for hydrogels with an increasing molar ratio. These hydrogels offer attractive tunability to meet certain criteria favorable to wound healing applications. The 4 wt% hydrogel with a molar ratio of 1:1 demonstrated adequate swelling behavior and mechanical properties suitable for soft tissue engineering, while the pore sizes and degradation time correlated with the requirements for regenerating skin tissue. These hydrogels further demonstrated very low cytotoxicity and good cell adhesion. The successful attainment of the objectives of this study, being on the controlled synthesis, preparation and characterization of the library of P(KA)/4PEG hydrogels, provides suitable motivation for further in vivo studies. For future work, it is recommended that the P(KA/4(PEG) hydrogels be tested on a selected animal wound model to determine the in vivo cytotoxicity and to evaluate the wound healing properties of the hydrogels.

## Figures and Tables

**Table 1:** A summary of the polymer concentrations of the P(KA) and 4-PEG-SG used in the preparation of the library of hydrogel solutions to achieve the total polymer concentrations of 1 wt%, 2 wt%, 4 wt%, 6 wt% and 8 wt%.

Total Polymer Concentration	Molar Ratio									
	1:1		2:1		4:1		8:1		16:1	
	P(KA)	4-PEG	P(KA)	4-PEG	P(KA)	4-PEG	P(KA)	4-PEG	P(KA)	4-PEG
**1 wt%**	0.16	0.84	0.28	0.72	0.44	0.57	0.61	0.39	0.76	0.24
**2 wt%**	0.32	1.68	0.56	1.44	0.87	1.13	1.21	0.79	1.51	0.49
**4 wt%**	0.65	3.36	1.11	2.89	1.74	2.26	2.42	1.58	3.02	0.98
**6 wt%**	0.97	5.03	1.67	4.33	2.61	3.39	3.64	2.36	4.53	1.47
**8 wt%**	1.29	6.71	2.22	5.78	3.48	4.52	4.85	3.15	6.04	1.96

**Table 2:** Polypeptide synthesized by ROP of NCA amino acids.

Entry	DP_exp_^a^	K/A Ratio ^a^	*M*_n Target_ (g·mol^−1^)	*M*_n NMR_ (g·mol^−1^)	*M*_n SEC_ (g·mol^−1^) ^b^	Đ ^b^	Free Amine (μmol·mg^−1^)
#1	79	0.63:0.37	18,342	14,862	20,928	1.33	2.55 ± 0.14
#2	96	0.60:0.40	18,342	17,608	26,933	1.18	2.02 ± 0.11

^a^ Degree of polymerization (DP) and K/A ratio determined from ^1^H NMR spectroscopy. ^b^ Number average molecular weight (*M*_n_) and dispersity (*Đ*) determined from SEC using PMMA as standard.

**Table 3:** Average pore sizes of the P(KA)/4-PEG hydrogels with varying molar ratios and total polymer concentrations.

Hydrogel	Average Pore Size (μm) ^a^		
	1:1	2:1	4:1
4 wt%	91 ± 39 ^b,c,k,l^	239 ± 68 ^b,d,n,o^	255 ± 51 ^c,d,q,r^
6 wt%	132 ± 58 ^e,f,k,m^	157 ± 48 ^e,g,n,p^	181 ± 64 ^f,g,q,s^
8 wt%	128 ± 55 ^h,i,l,m^	125 ± 45 ^h,j,o,p^	165 ± 67 ^i,j,r,s^

^a^ Calculated from 40 to 60 measurements from 3 or more images with size distributions approximating normal distributions. ^b–s^ The statistical *p*-values were obtained from Student’s *t*-tests between the experimental data with corresponding annotations, e.g., ^“b”^ indicates correlation between 4 wt% 1:1 and 4 wt% 2:1. The *p*-values are indicated below: ^b^ *p* < 0.001; ^c^ *p* < 0.001; ^d^ *p* = 0.086; ^e^ *p* = 0.014; ^f^ *p* < 0.001; ^g^ *p* = 0.021; ^h^ *p* = 0.377; ^i^ *p* = 0.001; ^j^ *p* < 0.001. ^k^ *p* = 0.050; ^l^ *p* = 0.119; ^m^ *p* = 0.333; ^n^ *p* < 0.001; ^o^ *p* < 0.001; ^p^ *p* < 0.001; ^q^ *p* < 0.001; ^r^ *p* < 0.001; ^s^ *p* = 0.116.

## Data Availability

The data presented in this study are available in the article.
